# Endogenous estrogen exposure and chronic kidney disease; a 15-year prospective cohort study

**DOI:** 10.1186/s12902-021-00817-3

**Published:** 2021-08-04

**Authors:** Maryam Farahmand, Fahimeh Ramezani Tehrani, Davood Khalili, Leila Cheraghi, Fereidoun Azizi

**Affiliations:** 1 Reproductive Endocrinology Research Center, Research Institute for Endocrine Sciences, Shahid Beheshti University of Medical Sciences, Tehran, Iran; 2 Prevention of Metabolic Disorders Research Center, Research Institute for Endocrine Sciences, Shahid Beheshti University of Medical Sciences, Tehran, Iran; 3 Department of Epidemiology and Biostatistics, Research Institute for Endocrine Sciences, Shahid Beheshti University of Medical Sciences, Tehran, Iran; 4 Endocrine Research Center, Research Institute for Endocrine Sciences, Shahid Beheshti University of Medical Sciences, Tehran, Iran

**Keywords:** Endogenous estrogen exposure, Chronic kidney disease (CKD), Menopause, Menarche, Estimated glomerular filtration rate (eGFR)

## Abstract

**Background:**

Despite strong evidence demonstrating the role of estrogen as a protective factor for kidney function in women, limited data are available regarding the influence of endogenous estrogen exposure (EEE) on chronic kidney disease (CKD). The present study aimed to assess the incidence of CKD in women with various levels of EEE.

**Methods:**

In a prospective population-based study over a 15-year follow-up, a total of 3043 eligible women aged 30–70 years, participating in Tehran-Lipid and Glucose-Study were recruited and divided into two groups (EEE < 11 and EEE ≥ 11 years). EEE calculated based on age at menarche, age at menopause, number and duration of pregnancies, lactation, and duration of oral contraceptive use after excluding the progesterone dominant phase of the menstrual cycle. Cox’s proportional hazards model was applied to estimate the hazard ratio of CKD between the study groups, after adjusting for confounders.

**Results:**

The total cumulative incidence rate of CKD was 50.1 per 1000 person years; 95% CI: 47.7–52.6); this was 53.9 (95%CI, 50.2–57.8) and 47.1 (95%CI, 44.0–50.4) per 1000 person years in women with EEE < 11 and EEE ≥ 11 years, respectively. The model adjusted for age, BMI, smoking, hypertension, and diabetes showed that the hazard ratio (HR) of incidence CKD in women with EEE < 11 compare to those with EEE ≥ 11 years in the subgroup of women aged< 45 years was 2.66(95% CI, 2.2, 3.2), whereas, in the subgroup aged ≥45 years, it was 1.22 (95% CI, 1.04, 1.4).

**Conclusion:**

This study shows a higher HR of CKD incidence in women with low EEE levels in their later life. Screening of these women for CKD may be recommended.

## Background

The kidney, a powerful endocrine organ, is an important modulator of endocrine function, and the main target for hormonal action [1]. Chronic kidney disease (CKD) can be determined as a persistent injury of the renal parenchyma which causes chronic deterioration of renal function that may progressively worsen to end-stage kidney disease (ESKD) [1].

Studies show that slower progression of CKD and lower incidence of ESKD in younger women compared to men, and omitting this gender protection after menopause, suggests a role for female hormones [2–4]. While the mechanisms responsible for the protection of kidneys by these hormones, mainly estrogen, are not completely understood, it seems to be due to induced vasodilation in the renal vessels, enhancing the production of nitric oxide (NO), attenuation of inflammation, and reduction in ischemia mediators [5–7].

It has been shown that estrogen stimulates the release of NO resulting in vasodilation; NO deficiency can be associated with acceleration of kidney injury by reduction of vasodilation and endothelial dysfunction. Estrogen also decreases the synthesis of renin and angiotensin-converting enzyme (ACE) and increases angiotensinogen synthesis [8]. Experimental studies demonstrate that administration of continuous estradiol can prevent glomerulosclerosis and albuminuria [9].

Despite existing strong evidence demonstrating the role of estrogen as a protective factor for kidney function in women, limited data are available regarding the influence of endogenous estrogen exposure (EEE) on chronic kidney disease (CKD); EEE can be calculated based on reproductive factors, including age at menarche, age at menopause, number and duration of pregnancies, lactation, and duration of oral contraceptive use [10]. In this prospective study, we aimed to investigate the incidence and hazard ratio of CKD among women with lower durations of EEE compared to those with higher exposure, after adjustment for known confounders.

## Methods

### Subjects & Study procedure

Subjects of the present study were recruited from among participants of the Tehran Lipid and Glucose Study (TLGS). This study is an ongoing prospective study, initiated in 1998, to determine the prevalence of non-communicable disease (NCD) risk factors among 15,000 participants aged > 3 years. This population was selected from the population of district 13 in Tehran using a multistage stratified cluster random sampling technique. This district was selected mainly due to the high rate of stability; moreover, its age distribution was representative of the overall population in Tehran. After recruitment, all study participants were followed in 3-year intervals. Demographic, habitus, reproductive history, family history, and cardio-metabolic risk factors were assessed through face to face interview at the initiation of the study and throughout the follow ups. A comprehensive physical exam including anthropometric measurements, systolic, and diastolic blood pressures was conducted at baseline and follow ups. Moreover, blood samples were drawn at each visit between 7:00 and 9:00 am after 12 h of overnight fasting for metabolic assessments. All blood analyses were performed at the TLGS research laboratory on the day of blood collection. The CKD status was identified at the initiation of the study and throughout the follow ups according to the Kidney Disease Outcome Quality Initiative guidelines (K/DOQI). The details of TLGS was published elsewhere [11].

There were 5226 women, regardless of menopausal status, aged 30–70 years selected for our study. After excluding those with CKD at baseline (*n* = 1425), those with missing data of CKD (*n* = 97), those with missing data on age at menarche (*n* = 1019), the number of remained women was 3141. We further excluded those with missing data for calculating EEE (*n* = 98), HRT users (*n* = 87), and those without at least one follow-up(*n* = 92). Finally, 3043 women remained for the present study.

**Fig. 1:**
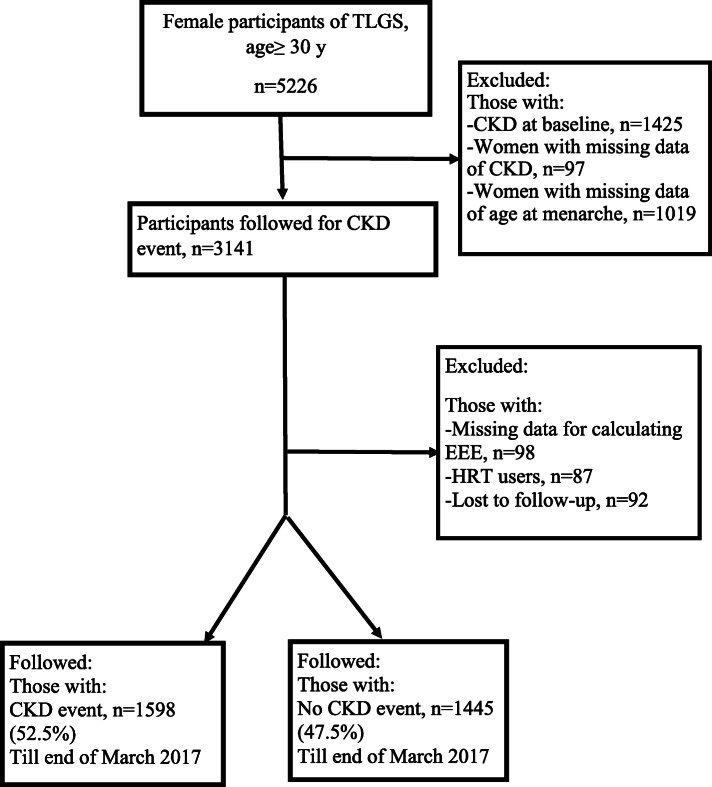
Study flowchart. Abbreviations: TLGS, Tehran Lipid and Glucose Study; CKD, Chronic Kidney Disease; EEE, Endogenous Estrogen Exposure; HRT, Hormone Replacement Therapy

The study flowchart is presented in Fig. 1.

### Measurements

Using a measuring tape with 0.5-cm accuracy, in a standing position against a wall, without shoes, and with shoulders in a normal position, height was measured. Using an electronic digital weighing scale with 0.1-kg accuracy, while women were minimally clothed, without shoes, weight was measured. Body mass index (BMI) was calculated using dividing weight in kilograms by height in meters squared.

Systolic and diastolic blood pressure (SBP and DBP) was measured twice after a 15 min rest in a sitting position on the right arm, and the mean was considered as the participants’ blood pressure. Weight was measured with individuals minimally clothed, using digital scales (Seca 707: range 0.1–150 kg) and recorded to the nearest 0.1 kg. Height was measured in a standing position, using a tape meter, while shoulders were in normal alignment.

Plasma glucose was measured using an enzymatic colorimetric method with glucose oxidase. Serum concentrations of creatinine (Cr) were tested by kinetic colorimetric Jaffe. The sensitivity of the assay was 0.2 mg/dL (range, 18–1330 μmol/L (0.2–15 mg/dL). Reference intervals based on the manufacturer’s recommendation was 53–97 μmol/L (0.6–1.1 mg/dL) in women. Intra-assay and inter-assay CVs were less than 3.1% at both baseline and follow-up phases. All biochemical assays were performed using commercial kits (Pars Azmoon Inc., Tehran, Iran) by a Selectra 2 autoanalyzer (Vital Scientific, Spankeren, The Netherlands) [12, 13].

### Definitions

Menopause was defined as the absence of menstruation during consecutive12 months according to the World Health Organization definition [14].

According to the Kidney Disease Outcome Quality Initiative guidelines (K/DOQI), CKD is defined as either kidney damage or Glomerular Filtration Rate (GFR) < 60 mL/min/1.73 m2 for > 3 months [15]. In the present study, we estimated GFR using the abbreviated prediction equation, provided by the Modification of Diet in Renal Disease (MDRD) study [16] as follows:

$$ \mathrm{GFR}=186\times {\left(\mathrm{SCr}\right)}^{-1.154}\times {\left(\mathrm{Age}\right)}^{-0.203}\times 0.742\times \left(0.742\ \mathrm{if}\ \mathrm{female}\right) $$

In this equation, estimated GFR (eGFR) is expressed as mL/min per 1.73 m2 and serum creatinine (SCr) is expressed as mg/dL; based on the guidelines, we considered CKD as an eGFR> 60 mL/min/1.73 m2, occurring at any time during the follow-up period.

Endogenous estrogen exposure (EEE) was defined as the time interval between age at menarche and menopausal age or age at CKD event or end of follow-up, whichever occurred earlier. To consider only E2 dominant of menstrual cycles, we omitted the cumulative durations of progesterone dominant phases of menstrual (only the first 2 weeks, follicular phase, for each menstrual cycle was considered) using as well as those of pregnancies and lactation (assumed 40 weeks for each birth or 20 weeks for each abortion); as a result, EEE is absolutely less than half of the time duration between menarche and menopause.

Hypertension was defined as systolic blood pressure (SBP)/ diastolic blood pressure (SBP) ≥ 140/90 mmHg or current treatment for diagnosed hypertension [17]. Diabetes was defined as fasting plasma glucose (FPG) ≥ 7.0 mmol/l or 2-h post 75 g glucose load ≥11.1 mmol/l or under current treatment for diagnosed diabetes [18]. Smoking status was categorized as ever smoker (current/past) or never smoker [19]. Physical activity was measured as the MET value multiplied by the duration of activity in minutes multiplied by the frequency of activity per week. Each activity was weighted via its relative power, referred to as a MET; one MET shows the energy spent for an individual at rest (1 MET = 3.5 ml/kg.min of oxygen consumption). Energy expending was estimated based on the metabolic equivalent, duration of the activity, and body weight. To get the total weekly leisure time energy expending the individual activities values were summed [20].

### Statistical analysis

Results are reported as mean and standard deviation (SD) for continuous variables and number (percentage) for categorical measures. For continuous variables with skewed distribution, the median (inter-quartile range) was calculated. Using the Cubic Spline regression approach, we assessed the knot as 11 years for EEE duration. Furthermore, participants were categorized into two groups according to cut-offs of EEE duration, of< 11, and ≥ 11 years. Also, the analysis was repeated by age group < 45 and ≥ 45 years at baseline.

Time to event was specified as a time of censoring or date of incidence of CKD or age at menopause, whichever happened first. Participants were censored as a result of death, loss to follow-up, or the end of the observation duration. For censored subjects or for those moving out of the area and thus lost to follow-up, the most recent follow-up visit was used. “Time” was considered as the interval between the first and the last follow-up dates. The event date for CKD was defined as midtime between the dates of the follow-up visit at which the CKD was recognized for the first time, and the last follow-up visit before diagnosis. The incidence rate of CKD was calculated per 1000 person years of follow up between those with EEE < 11 and EEE ≥ 11 years. Cumulative incidence of CKD was measured via the Kaplan-Meier method and compared between these 2 groups, using the log-rank statistic.

Cox proportional hazards regression model was used to estimate the hazard ratio (HR) and 95% confidence interval (CI) for the group with EEE < 11 years compared to those with EEE ≥ 11 years. The Cox model was adjusted for age, BMI, smoking, hypertension, and diabetes. The proportional hazards assumption of the Cox models was assessed graphically and was satisfied. The Statistical Package for Social Sciences (SPSS version 20; SPSS Inc.) and STATA software (version 13; STATA Inc.) was used for data analysis.

## Results

Characteristics of the study subjects according to the EEE cutoff value of 11 years are shown in Table 1. Mean ± SD age and menarcheal age of study subjects were 43.1 ± 10.1, and 13.6 ± 1.5 years, respectively.

**Table 1:** Characteristics of subjects in groups by the cut-off of exposure durations of endogenous estrogen

	Duration of endogenous estrogen exposure		
Variables	Group 1 < 11 years	Group 2 ≥11 years	Total
Subjects (no)*	1429 (47.0)	1614(53.0)	3043(100)
Age(years) **	39.2 ± 8.8	46.6 ± 9.8	43.1 ± 10.1
Menarcheal age(years) **	13.7 ± 1.4	13.5 ± 1.5	13.6 ± 1.5
Body mass index (kg/m^2^) **	28.1 ± 4.6	28.5 ± 4.8	28.3 ± 4.7
Hypertension (yes)*	195(13.8)	425(26.8)	620(20.6)
Antihypertensive drug use *	60(4.2)	150(9.3)	210(6.9)
Angiotensin-converting-enzyme inhibitor use*	9 (0.6)	19(1.2)	28(0.9)
Diabetes type 2*	110(7.9)	238(15.1)	348(11.7)
Anti-diabetes type 2 drug use*	39(2.7)	92(5.7)	131(4.3)
Ever Smoker (yes) *	82(5.8)	72(4.5)	154(5.1)
eGFR (ml/min per1.73 m^2^)	71.9 ± 8.5	70.7 ± 8.0	71.3 ± 8.3
Creatinine (mg/dl)**	0.94 ± 0.08	0.92 ± 0.08	0.93 ± 0.06
Physical activity(MET- min/week)**	1639 ± 2350.5	1577.7 ± 2049.4	1606.7 ± 2195.2
Menopause status (yes)*	345(24.1)	1420(88.0)	1765(58)
Menopausal age(years) **	44.1 ± 6.6	50.0 ± 4.0	48.8 ± 5.2
Total duration of pregnancies (years)**	2.72 ± 1.4	3.09 ± 1.5	2.90 ± 1.5
Total duration of hormonal contraceptive use (years) **	0.55 ± 1.0	0.47 ± 0.5	0.53 ± 0.9
Total duration of breastfeeding (weeks) **	4.35 ± 2.6	2.25 ± 1.6	3.7 ± 2.5
Follow-up time (years)***	15.5(12.2,16.5)	15.6(12.5,16.6)	15.6(12.4,16.6)

Note: ANOVA test, Mann-Whitney test, and Chi-square test were used as appropriate

* Number and percentage

** Mean ± SD

***Median (Interquartile range)

Note: eGFR estimated glomerular filtration rate; Body mass index& age are presented at baseline; Total duration of hormonal contraceptive use was calculated only in contraceptive users. Menopause status was assessed during follow-up or before event or censoring. Menopausal age was calculated among participants who had reached menopause. MET, metabolic equivalent task

Note: Group1: Endogenous estrogen exposure duration < 11 years, group, 2: Endogenous estrogen exposure duration ≥11 years

The median and 25–75%follow-up time for the current analysis was 15.6 and 12.4, 16.6 years. The overall incidence of CKD was 50.1 per 1000 person years (95%CI: 47.7–52.6). It was 53.9(95%CI: 50.2–57.8), and 47.1(95%CI: 44.0–50.4) per 1000 person years in women with EEE < 11 and EEE ≥ 11 years, respectively. As a result of subgrouping the participants into two age groups (< 45, and ≥ 45 years), in those aged< 45, the incidence of CKD was 45.6 (95%CI: 41.9, 49.7) per 1000 person years in women with EEE < 11 years. In the recent age group, the incidence of CKD was 22.4(95%CI: 19.6, 25.5) per 1000 person years in women with EEE ≥ 11 years. In those aged ≥45, the incidence of CKD was 92.5 (95%CI: 81.3, 105.2) per 1000 person years in women with EEE < 11 years. In the same age group, the incidence of CKD was 78.9 (95%CI: 72.9, 85.5) per 1000 person years in women with EEE ≥ 11 years (Table 2).

**Table 2:** Unadjusted and multiple adjusted hazard ratios of incident CKD by cut off value of 11 years for the duration of EEE and subgroups of age at baseline

Endogenous estrogen exposure duration	Crude number of incident CKD (incident rate per 1000 person years) (95% CI)	Unadjusted		Adjusted*	
		HR (95% CI)	***P*** value	HR (95% CI)	***P*** value
**Total**					
***N*** **= 3043**					
Group1 (< 11 years)	769 (53.9)	1.17 (1.1,1.3)	0.002	4.0 (2.5,6.3)	< 0.001
*N* = 1429	(50.2,57.8)				
Group 2 (≥11 years)	829 (47.1)	Ref	Ref	Ref	Ref
*N* = 1614	(44.0,50.4)				
**< 45 years**					
***N*** **= 1860**					
Group1 (< 11 years)	537 (45.6)	2.2 (1.9,2.5)	< 0.001	2.7 (2.2,3.2)	< 0.001
*N* = 1120	(41.9,49.7)				
Group 2 (≥11 years)	222 (22.4)	Ref	Ref	Ref	Ref
*N* = 740	(19.6,25.5)				
**≥45 years**					
***N*** **= 1183**					
Group1(< 11 years)	232 (92.5)	1.17 (1.0,1.4)	0.03	1.22 (1.04,1.4)	0.01
*N* = 309	(81.3105.2)				
Group 2 (≥11 years)	607 (78.9)	Ref	Ref	Ref	Ref
*N* = 874	(72.9,85.5)				

Note: CI, Confidence interval; HR, Hazard ratio

Abbreviations: *CKD* chronic kidney disease, *EEE* endogenous estrogen exposure

*Adjusted for baseline age and body mass index, smoking, hypertension, diabetes type 2

**Fig. 2:**
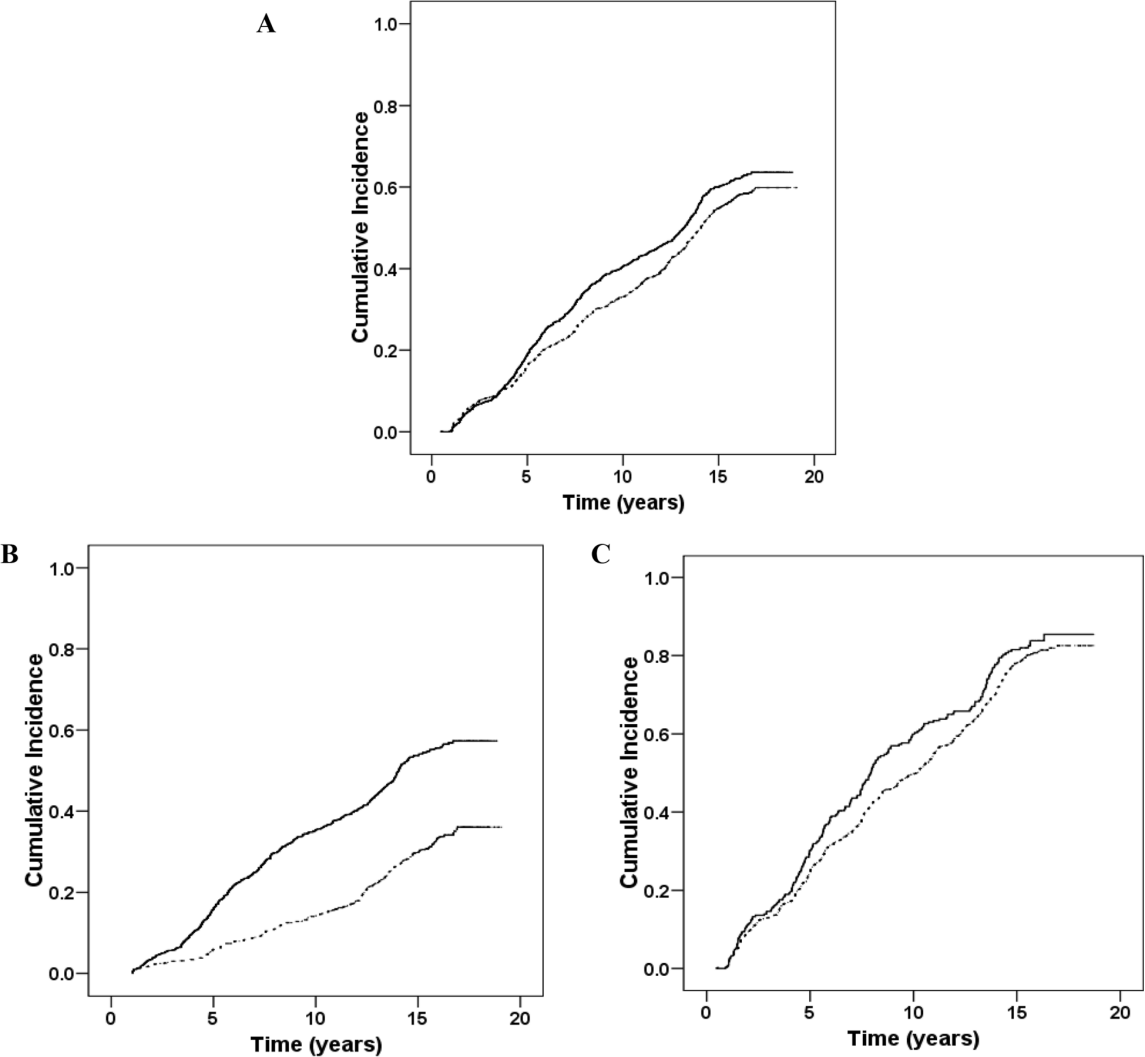
Kaplan-Meier cumulative estimates of incidence rates of CVD in subjects, according to the cutoff of endogenous estrogen exposure duration. A: Total of participants; B: Participants aged < 45 years; C: Participants aged≥45 years. Note: Endogenous estrogen exposure duration< 11 years (solid line), and Endogenous estrogen exposure duration≥11 years (dotted line)

Kaplan-Meier cumulative incidence of CKD was statistically different between women with EEE < 11 and those with EEE ≥ 11 years, among total (*p* = 0.002) and two age groups (*p* < 0.001 and *p* = 0.03) (Fig. 2).

As the interaction between age groups and EEE duration was statistically significant (*P* < 0.001); so the Cox regression model was conducted separately in age groups. In the unadjusted model, the hazard of CKD in women with EEE < 11 years was 17%more than those women with EEE ≥ 11 years (95% CI: 1.1,1.3; *p* = 0.002). The adjusted model showed that the hazard of CKD incidence in women with EEE < 11 years compared to those with ≥11 years was 4.0 fold (95% CI: 2.5, 6.3; *p* < 0.001).

In the subgroup of women aged < 45, the hazard of CKD incidence in women with EEE < 11 years compared to those women with EEE ≥ 11 years was 2.2 fold (95% CI:1.9,2.5; p < 0.001), in the unadjusted model. This HR is changed to 2.7(95%CI:2.2,3.2; p < 0.001) after adjustment for age, BMI, smoking, hypertension, and diabetes.

In the subgroup of women aged ≥45, the hazard of CKD incidence in women with EEE < 11 years was 17% (HR:1.17; 95% CI:1.0,1.4; *p* = 0.03) more than those women with EEE ≥ 11 years, in the unadjusted model. This HR is changed to 1.22(95% CI: 1.04,1.4; *p* = 0.01) after adjustment for the above-mentioned variables (Table 2).

## Discussion

To the best of our knowledge, this is the first study reporting the effects of endogenous estrogen exposure on chronic kidney disease We found that after adjustment for all influencing variables, the hazard ratio of CKD among those women with > 11 years of EEE is fourfold (95% CI: 2.5,6.3, *P* < 0.001) that of those with ≥11 years of exposure. An adverse effect even stronger among those women aged< 45 2.7(95% CI: 2.2, 3.2) vs. 1.22(1.04, 1.4) in women aged≥45 years was observed.

There is controversy about the impact of sex hormones on renal function and disease as well as the gender dependency of CKD; the lower susceptibility of women could be due to the absence of testosterone or the presence of estrogen [21] via elevated synthase production nitric oxide in kidneys [22–24].

It has been shown that estrogen may affect kidney function through several pathways including improved metabolism, the selectivity of Angiotensin Type 2 (AT2) receptor signaling, diminished oxidative stress, and differential renin-angiotensin system (RAS) [25]. There is some evidence that NO deficiency can be associated with acceleration of renal injury, based on receptors that impaired vasodilation and endothelial dysfunction have been reported in CKD [5].

Estradiol may improve kidney function by suppressing the transforming growth factor β − 1 (TGF- β − 1) that induces mesangial cell apoptosis [26], and enhancing mesangial cell growth [27] by up-regulating NO synthase activity via vascular agents; furthermore, estrogen receptor α that largely expressed in the kidneys [28], has the opposite effect of apoptosis in podocytes [29] and also acts as a regulator of renal sodium and potassium homeostasis and the renin-angiotensin pathway [30]. Several studies have reported the anti-apoptotic and anti-fibrotic effects of endogenous estrogens in the kidney [2, 31–33], which may partly explain the protective effect of estrogen on the kidney. Estrogen may also have kidney protective effects by reducing renin and angiotensin-converting enzyme (ACE) synthesis and increasing angiotensinogen synthesis [8]. Also, estrogen acts as a mediator by reducing arterial pressure via signaling the AT2 receptor and ACE2 [25]. Animal studies reveal the acceleration of progression of glomerulosclerosis due to estrogen shortage, resulting in diminished glomerular permeability and ischemia-reperfusion damage [2, 31–33].

Endogenous estrogen has been introduced as an important influential factor in NCDs (such as CKD) for its promotion of angiogenesis and vasodilation and decreasing of reactive oxygen species, oxidative stress, and fibrosis [25]. Shorter durations of EEE have been reported to be associated with increased risk of osteoporosis, total mortality, venous thromboembolism, and CVD [10, 19]. However, to the best of our knowledge, the association between EEE and CKD has not been reported before.

We found that the impact of the duration of EEE on CKD among younger women is even stronger than in older counterparts, an important finding with high clinical utility given the higher morbidity and mortality of younger women with CKD [35].In addition to early menopause or late age at menarche, other physiological situations such as increased parity and longer duration of lactation may result in a decrease of EEE. Furthermore, pathologic causes of low estrogen levels in young women including excessive exercise, eating disorders, and low-functioning pituitary gland needs to be considered [34, 36–38].

However, with aging, this association weakens, possibly due to overexpression of other known risk factors of CKD such as diabetes and hypertension [39].

This study is strengthened by the use of a comprehensive population-based data set with a long follow-up, large sample size, and precise estimation of CKD and influential factors, which enabled us to perform survival analysis**,** and adjust for the most important potential confounders; however, it does have some limitations. The main one is, as in most epidemiologic studies [1, 40], we have not repeated Cr measurements within 3 months to confirm a chronic reduction in GFR. Second, recall bias for some components that have been used for calculation of EEE; however, repeating these measurements every 3 years may reduce the risk of this bias. Although we tried to adjust our results for major known confounders for which data were available, other potentially influencing factors such as diet have not been considered.

## Conclusion

Our findings showed that a lower duration of endogenous estrogen exposure especially among reproductive age women can be considered as a risk factor for CKD, and the early diagnosis of this susceptible group may improve their short-term and long-term morbidities.

## Data Availability

The datasets generated and/or analyzed during the current study are not publicly available due to confidentiality considerations.

## Abbreviations

EEE: Endogenous estrogen exposure
CKD: Chronic kidney disease
eGFR: estimated glomerular filtration rate
ESKD: End-stage kidney disease
NO: Nitric oxide
ACE: Angiotensin-converting enzyme
TLGS: Tehran Lipid and Glucose Study
NCD: Non-communicable Disease
CVD: Cardiovascular disease
SCr: Serum creatinine
K/DOQI: Kidney Disease Outcome Quality Initiative guidelines
MDRD: Modification of Diet in Renal Disease
FPG: Fasting plasma glucose
SBP: Systolic blood pressure
DBP: Diastolic blood pressure
SD: Standard deviation
CI: Confidence interval
AT2: Angiotensin Type 2
RAS: Renin-angiotensin system
